# Latent Tuberculosis Infection among a Large Cohort of Medical Students at a Teaching Hospital in Italy

**DOI:** 10.1155/2015/746895

**Published:** 2015-02-01

**Authors:** Paolo Durando, Cristiano Alicino, Andrea Orsi, Ilaria Barberis, Chiara Paganino, Guglielmo Dini, Giovanni Mazzarello, Valerio Del Bono, Claudio Viscoli, Francesco Copello, Dimitri Sossai, Giovanni Orengo, Laura Sticchi, Filippo Ansaldi, Giancarlo Icardi

**Affiliations:** ^1^Department of Health Sciences, University of Genoa and Hygiene and Infection Control Unit, IRCCS AOU San Martino-IST teaching Hospital, Largo R. Benzi 10, 16132 Genoa, Italy; ^2^Postgraduate School in Occupational Medicine, University of Genoa and Occupational Medicine Unit, IRCCS AOU San Martino-IST teaching Hospital, Largo R. Benzi 10, 16132 Genoa, Italy; ^3^Department of Health Sciences, University of Genoa and Infectious Diseases Unit, IRCCS AOU San Martino-IST teaching Hospital, Largo R. Benzi 10, 16132 Genoa, Italy; ^4^Health Safety and Prevention Unit, IRCCS AOU San Martino-IST teaching Hospital, Largo R. Benzi 10, 16132 Genoa, Italy; ^5^Quality and Risk Management Unit, IRCCS AOU San Martino-IST teaching Hospital, Largo R. Benzi 10, 16132 Genoa, Italy

## Abstract

The surveillance of latent tuberculosis infection (LTBI) in both healthcare workers and healthcare students is considered fundamental for tuberculosis (TB) prevention. The aim of the present study was to estimate LTBI prevalence and evaluate potential risk-factors associated with this condition in a large cohort of medical students in Italy. In a cross-sectional study, performed between March and December 2012, 1511 eligible subjects attending the Medical School of the University of Genoa, trained at the IRCCS San Martino-IST Teaching Hospital of Genoa, were actively called to undergo the tuberculin skin test (TST). All the TST positive cases were confirmed with an interferon-gamma release assay (IGRA). A standardized questionnaire was collected for multivariate risk analysis. A total of 1302 (86.2%) students underwent TST testing and completed the questionnaire. Eleven subjects (0.8%) resulted TST positive and LTBI diagnosis was confirmed in 2 (0.1%) cases. Professional exposure to active TB patients (OR 21.7, 95% CI 2.9–160.2; *P* value 0.003) and previous BCG immunization (OR 28.3, 95% CI 3.0–265.1; *P* value 0.003) are independently associated with TST positivity. Despite the low prevalence of LTBI among Italian medical students, an occupational risk of TB infection still exists in countries with low circulation of *Mycobacterium tuberculosis*.

## 1. Introduction

A work-related risk of latent tuberculosis infection (LTBI) and active tuberculosis (TB) among healthcare workers (HCWs) exists even in areas with low incidence of TB among the general population, such as Europe [1–3]. The majority of occupational active cases in the healthcare sector occur when the TB infection risk is underestimated and control programs are lacking [4, 5]. Moreover, improving the knowledge about TB transmission and adopting effective control measures to face the spread of the infection in healthcare setting have been recommended with the aim of reducing the risk of nosocomial infection [6–8].

Medical students attending teaching hospitals could be exposed to similar occupational risks as HCWs. Therefore, screening for LTBI of these categories, for an early diagnosis of cases and preventing progression to active disease, represents a fundamental aspect of hospital infection control programs and is recommended also in low-incidence TB countries, including Italy [9–11].

Several surveys have been performed among healthcare students in countries with a high incidence of TB, reporting high LTBI prevalence figures, widely ranging from 6.9% to 72% [13–18]; however, very few studies have investigated the epidemiology of TB infection and the associated risk factors among undergraduate students in areas with a low incidence of TB [19–21]. In a recent survey conducted by our research group, on more than 700 healthcare students in Genoa, Italy, a low prevalence of TST positivity (1.4%) was reported, and the only significant association with this condition was to be born in high TB incidence areas [21]. Due to some limits reported in this study, particularly the lack of information about previous exposure to active TB cases, at either a professional or community level, and the different type of clinical training between medical, nursing, and midwifery students, which did not permit an accurate risk assessment for TB infection, another survey, which included a larger study population, targeted only medical students, and improved the accuracy of the risk analysis, was performed to gain further insights into the epidemiology of TB infection in this risk group.

## 2. Methods

### 2.1. Study Design

A cross-sectional survey, using routine demographic, clinical, and laboratory data, was performed.

### 2.2. Setting and Study Population

The study was carried out between March and December 2012 and it involved all the medical students attending the Medical School of the University of Genoa, trained at the IRCCS AOU San Martino-IST Teaching Hospital of Genoa, Italy, the regional tertiary adult acute care reference hospital with 1,400 beds. Almost all the cases of infectious TB that occur in the Liguria Region, where Genoa is located, are hospitalized at the Infectious Diseases Unit of the IRCCS San Martino-IST Teaching Hospital, since it is the only facility equipped with negative-pressure rooms for contagious patients.

Approximately 250 students were annually admitted to the Medical School of the University of Genoa during the last decade: all the 1511 students attending the six years of the training program at the time of the investigation were actively summoned to undergo TST and fill in a standardized questionnaire. Students were classified as “preclinical” (from the 1st to the 3rd year of the Medical School program), with no contact with patients inside the hospital, and “clinical” (from the 4th to 6th year), attending various medical, laboratory, and surgical departments of the hospital, including the infectious disease wards.

### 2.3. Tuberculin Skin Testing Technique

Trained HCWs applied TST using the Mantoux technique. A standard dose of 0.1 mL of purified protein derivative (PPD-Rt 23, 2 Tubercoline Units, Staten Serum Institut, Copenaghen, Denmark) was slowly injected intradermally into the inner surface of the forearm and a small, blanched papule with a diameter of 8–10 mm appeared, disappearing after approximately ten minutes. The skin was slightly stretched, and the needle held almost parallel to the skin surface with the bevel facing upwards. The injection was made with a plastic disposable syringe, with a short bevel needle (25-gauge). The reaction was read by measuring the diameter of induration across the forearm (perpendicular to the long axis) in millimeters. An induration ≥10 mm in healthy subjects was considered positive. The skin test reaction should be read between 48 and 72 hours after administration.

### 2.4. Management of TST-Positive Students

TST-positive cases were also tested with an interferon-gamma release assay (IGRA; QuantiFERON TB-Gold Cellestis, Carnegie, Australia), to confirm the diagnosis of LTBI, as also recommended in other countries [22–24]. Indeed, it is well known that IGRA has major specificity compared to conventional TST and that IGRA positivity should not be attributed to boosting induced by a previous TST [22, 25].

All the IGRA positive cases were carefully examined by an infectious diseases specialist and underwent chest radiography. Moreover, clinical signs and symptoms of active TB were illustrated to these subjects, and they were strongly recommended to immediately report their probable onset to the Occupational Health Unit and the Infection Control team of the hospital.

### 2.5. Data Collection

A standardized questionnaire was administered to all students included in the survey.

Information about age, gender, nationality, birth in a high or low TB incidence country, past and recent medical history, current health status, year of attendance at the Medical School,* Bacille Calmette-Guérin* (BCG) vaccination history, and exposure to active TB cases both at a professional (inside and outside the teaching hospital) and at community level (i.e., family, social activity) was obtained.

### 2.6. Ethics

All the activities of the study were performed in compliance with the current healthcare standards according to the recommendations of the Italian Ministry of Health and the Declaration of Helsinki [11, 26]. According to Italian legislation concerning guidelines on observational studies, ethical approval for conducting this survey was unnecessary, and on this basis, cross-sectional studies do not require a formal approval by local institutional review boards [27]. However, the study was regularly notified to the Ethics Committee of the IRCCS AOU San Martino-IST Teaching Hospital of Genoa, Italy. Eligible subjects were informed by a physician about the rationale and aims of the survey and all those who were included provided a written informed consent; personal information was protected according to Italian law [28]. The study was included in the “2012-2013 Risk Assessment Management Program” of the IRCCS AOU San Martino-IST Teaching Hospital.

## 3. Statistical Analysis

All the information collected through the questionnaire and the TST results were entered and analyzed using Epi-Info 7.0 (Centers for Disease Control and Prevention, CDC, Atlanta, GA, USA). Additional analyses were carried out using the SPSS Statistics version 20.0 (IBM Corp., Armonk, NY, USA). Association between categorical variables and the main outcome of interest, TST positivity, were tested using the Chi-squared test or Fisher exact test. All the categorical variables associated with TST positivity (*P* ≤ 0.1) were added to a multivariate logistic regression analysis to identify independent variables associated with TST positivity; furthermore a nested multivariate approach was used to study the possible confounding role of some variables.

## 4. Results

From March to December 2012, 1302 (86.2%) out of the 1511 eligible students performed TST screening, according to the procedures described above, and properly completed the questionnaire. Two hundred and nine (13.8%) students did not participate. The main demographic and epidemiological characteristics of the study population are outlined in Table 1 [12]. The majority of the students were born in Italy (1226/1302, 94.2%), with a mean (SD) age of 22.4 (2.4) years. Only 21 (1.6%) subjects enrolled in the survey were born in a country characterized by a high TB incidence (i.e., ≥20 cases per 100,000 inhabitants yearly) [12]. Nearly half (610/1302, 46.8%) of the study population was exposed to patients during the clinical training program, and 76 (5.8%) students reported a previous exposure to infectious TB cases. In particular, nearly 5% of the students reported to have a previous professional contact with active TB cases. BCG immunization had been previously performed in only 47 (3.6%) out of the 1302 students; nine (42.9%) out of 21 students born in countries at a high TB incidence received BCG immunization.

The proportion of positivity to TST was 0.8% (11/1302). Four out of 11 TST-positive subjects were not immunized with BCG: all these students were Italian and 2 reported a previous professional contact with a case of infectious TB. These last 2 students also resulted positive by IGRA and LTBI was diagnosed. Accurate clinical examination, together with a chest radiography, was performed in the individuals with a positive IGRA response and resulted negative, confirming the good health conditions of the infected students.

TST positivity was statistically associated with being a foreign student (9.2% versus 0.3%; *P* value <0.0001), with birth in a high TB incidence country (14.3% versus 0.6%; *P* value = <0.0001), with a previous vaccination with BCG (14.9% versus 0.3%; *P* value <0.0001), and with previous professional exposure to active TB cases (3.2% versus 0.7%; *P* value = 0.04) (Table 2). TST positivity seemed to be also associated with clinical level of training (1.2% versus 0.6%; *P* value = 0.3), even if the difference were not statistically significant.

At multivariate analysis previous BCG immunization (OR 28.3, 95% CI 3.0–265.1; *P* value = 0.003) and professional exposure with active TB cases (OR 21.7, 95% CI 2.9–160.2; *P* value = 0.003) were the only conditions that resulted independently associated with TST positivity (Table 3).

With nested approach, previous professional exposure with active TB cases (*P* value = 0.04) and birth in a high TB incidence country without previous vaccination with BCG (*P* value = <0.0001) resulted independently associated with TST positivity.

## 5. Discussion

Programs for the screening and treatment of LTBI cases in HCWs and undergraduate healthcare students, together with other interventions aimed at reducing the risk of transmission, represent fundamental tools of TB control programs in the healthcare setting. These activities are recommended in many countries, such as Italy, where an annual TB incidence of 4.9 per 100,000 inhabitants has been estimated [7, 10, 11, 22].

To the best of our knowledge, this is the first study that investigated both the prevalence and the potential risk factors associated with LTBI in a large cohort of medical students trained at a reference teaching hospital in a country with a low TB incidence.

In our survey, a very low prevalence of TST-positive cases (0.8%) resulted among medical students and a diagnosis of LTBI was confirmed by IGRA testing in only two (0.1%) cases. These data confirm the findings of a previous survey by our research group, performed on nearly 700 healthcare students attending the Medical, Nursing, and Midwifery Schools of the University of Genoa, where the prevalence of TST positives and of LTBI confirmed cases resulted 1.4% and 0.5%, respectively. The few studies performed in areas with a low circulation of* Mycobacterium tuberculosis *further confirm this epidemiological picture of LTBI among healthcare trainees [19, 20, 29]. In particular, Schablon and colleagues performed epidemiological surveys by screening undergraduate students and young professionals for LTBI in Germany, where a TB incidence of 5.3 cases per 100,000 population was reported in 2011 [30]. These surveys were performed in small samples using IGRA testing, which is known to have a major specificity compared with conventional TST [22]. In the first prevalence study, performed between 2005 and 2009, they found that none of the 110 trainees or young professionals included in the study showed positive results [19]. In another prospective cohort survey performed between 2008 and 2009, the prevalence of LTBI among 194 students at the beginning of their training, as healthcare worker or nurse, was 2.1% [20], while a figure of 3.5% was reported in another study among German HCWs under 30 years old [29].

The proportion of TST positivity resulted twice among clinical students compared with preclinical, even if the difference were not statistically significant. Moreover a previous professional exposure to active TB cases resulted independently associated with TST positivity, at both multivariate and nested analysis. Even more interestingly, the only two confirmed cases of LTBI in the study sample reported this specific risk-condition. In the above-mentioned prospective study performed in Germany [20], an annual follow-up with IGRA testing was planned for the trainees during each year of the professional program, but none of them, who reported having a contact with infectious TB patients, were found positive at the end of the three-year long survey [20]: our results, even if obtained in a cross-sectional study, seem to differ from the German experience.

Despite the fact that the risk analysis in our study needs to be interpreted carefully, because of the small number of TST-positive cases, the association between professional exposure to active TB cases and TST positivity suggests some useful recommendations for prevention of TB in the healthcare setting. First, the proper adoption of individual protection devices and control measures needs to be stressed among healthcare students in teaching hospitals, even in countries at low TB incidence, to reduce the professional risk of transmission from infectious patients. Moreover, students exposed to active TB patients need to undergo follow-up with TST for rapid identification of skin conversion to improve the control of nosocomial infection: in this view, the collection of a baseline TB immunological status is fundamental to demonstrate the acquisition of the LTBI during the training activity.

Finally, even if a previous vaccination with BCG was the only other independent variable associated with TST positivity at multivariate analysis, also birth in a high TB incidence country without previous immunization resulted associated at nested approach. These findings are consistent with other experiences [19]. Not surprisingly, immunization was rarely recorded in the study sample (3.6%), in consistency with the current Italian guidelines for TB prevention that recommend vaccination of HCWs and students only in selected cases, based on risk assessment at hospital level (i.e., individuals with unavoidable exposure to highly contagious multidrug-resistant TB cases and individuals with contraindications to LTBI preventive treatment) [11].

This study has some limits. The main one is the cross-sectional study design: for this reason, changes over time could not be monitored and this could impact on the accuracy of risk analysis. Moreover, a single-step TST procedure was used, although IGRA testing was systematically carried out in the event of TST positivity, thus increasing the specificity of the confirmed diagnosis of LTBI. Additionally, there was a lack of demographic and epidemiological information concerning the students who refused to take part in the survey.

## 6. Conclusions

Our results support the evidence that despite the fact that the prevalence of LTBI is very low among undergraduate medical students in countries with a low TB incidence and with high in-hospital hygiene standards, an occupational infection risk of TB still exists due to professional exposure to active TB patients. This scenario confirms current recommendations for systematic screening of all healthcare students before the clinical training. Larger studies are needed to confirm our findings to guarantee an optimal control of TB in the healthcare setting of countries with a low circulation of* Mycobacterium tuberculosis*.

## Figures and Tables

**Table 1:** Demographic, epidemiological, and clinical characteristics of a cohort of medical students (*n* = 1302) trained at a regional tertiary adult acute care reference hospital in Italy.

Variables	*N*	%
Total number of participants in the study	**1302**	**100**
Mean age, year (SD)	22.4 (2.4)	
Gender		
Male	560	43.0
Female	742	57.0
Nationality		
Italian	1226	94.2
Foreign	76	5.8
Born in a high TB incidence country^*^		
No	1281	98.4
Yes	21	1.6
Year of attendance		
First	219	16.8
Second	287	22.0
Third	186	14.3
Fourth	262	20.1
Fifth	183	14.1
Sixth	165	12.7
Level of training		
Preclinical	692	53.2
Clinical	610	46.8
BCG immunization		
No	1255	96.4
Yes	47	3.6
Previous exposure to active TB case		
No	1208	92.8
Household	13	1.0
Professional	63	4.8
Unknown	18	1.4
TST result		
Negative	1291	99.2
Positive	11	0.8

SD: standard deviation.

TB: tuberculosis.

BCG: Bacille Calmette-Guérin.

TST: tuberculin skin testing.

^*^High incidence: ≥20 cases per 100,000 population [12].

**Table 2:** Proportion of positive tuberculin skin testing response stratified by the main collected variables.

Variables	TST-positive result	%	*P* value
Gender			
Male	5/560	0.9	0.9
Female	6/742	0.8	
Nationality			
Italian	4/1226	0.3	<0.0001
Foreign	7/76	9.2	
Born in a high TB incidence country^*^			
No	8/1281	0.6	<0.0001
Yes	3/21	14.3	
Year of attendance			
First	2/219	0.9	0.6
Second	2/287	0.7	
Third	0/186	0	
Fourth	4/262	1.5	
Fifth	1/183	0.5	
Sixth	2/165	1.2	
Level of training			
Preclinical	4/692	0.6	0.3
Clinical	7/610	1.2	
BCG immunization			
No	4/1255	0.3	<0.0001
Yes	7/47	14.9	
Previous professional exposure to active TB case			
No	9/1221	0.7	0.09
Yes	2/63	3.2	

SD: standard deviation.

TB: tuberculosis.

BCG: Bacille Calmette-Guérin.

TST: tuberculin skin testing.

^*^High incidence: ≥20 cases per 100,000 population [12].

**Table 3:** Association between a positive tuberculin skin testing response and potential independent variables: results of multivariate logistic regression.

Variables	Multivariate analysis	
	OR (95% CI)	*P* value
Nationality		
Foreign/Italian	5.0 (0.5–45.4)	0.16
Born in a high TB incidence country^*^		
Yes/no	2.3 (0.4–12.7)	0.35
BCG immunization		
Yes/no	28.3 (3.0–265.1)	0.003
Professional exposure to active TB case		
Professional exposure/no professional exposure	21.7 (2.9–160.2)	0.003

OR: odds ratio.

95% IC: 95% confidence interval.

TB: Tuberculosis.

BCG: Bacille Calmette-Guérin.

^*^High incidence: ≥20 cases per 100,000 population [12].
